# Pleiotropic Functions of Cytochrome P450 Monooxygenase-Derived Eicosanoids in Cancer

**DOI:** 10.3389/fphar.2020.580897

**Published:** 2020-10-29

**Authors:** Ying Luo, Jun-Yan Liu

**Affiliations:** ^1^ Department of Clinical Laboratory, Changning Maternity and Infant Health Hospital, East China Normal University, Shanghai, China; ^2^ Center for Novel Target & Therapeutic Intervention, Institute of Life Sciences, Chongqing Medical University, Chongqing, China

**Keywords:** CYP = cytochrome P450, eicosanoid, cancer, morphism, mechanism

## Abstract

Eicosanoids are a class of functionally bioactive lipid mediators derived from the metabolism of long-chain polyunsaturated fatty acids (PUFAs) mediated by multiple enzymes of three main branches, including cyclooxygenases (COXs), lipoxygenases (LOXs), and cytochrome P450s (CYPs). Recently, the role of eicosanoids derived by COXs and LOXs pathways in the control of physiological and pathological processes associated with cancer has been well documented. However, the role of CYPs-mediated eicosanoids, such as epoxyeicosatrienoic acids (EETs), epoxyoctadecenoic acids (EpOMEs), epoxyeicosatetraenoic acids (EpETEs), and epoxydocosapentaenoic acids (EDPs), as well as hydroxyeicosatetraenoic acids (HETEs), in tumorigenesis and cancer progression have not been fully elucidated yet. Here we summarized the association of polymorphisms of CYP monooxygenases with cancers and the pleiotropic functions of CYP monooxygenase-mediated eicosanoids (EETs, EpOMEs, EpETE, EDPs, and 20-HETE) in the tumorigenesis and metastasis of multiple cancers, including but not limited to colon, liver, kidney, breast and prostate cancers, which hopefully provides valuable insights into cancer therapeutics. We believe that manipulation of CYPs with or without supplement of ω-3 PUFAs to regulate eicosanoid profile is a promising strategy to prevent and/or treat cancers.

## Introduction

Eicosanoids, a class of bioactive lipid mediators, are the metabolites of long-chain n-3 and n-6 polyunsaturated fatty acids (PUFAs) mediated by three primary enzymatic systems, cyclooxygenases (COXs), lipoxygenases (LOXs), and cytochrome P450s (CYPs) enzymes. The common PUFAs include arachidonic acid (20:4 n = 6, AA), linoleic acid (18:2 n = 6, LA), γ-linolenic acid (18:3 n = 6, GLA), α-linolenic acid (18:3 n = 3, ALA), eicosatetraenoic acid (20:5 n = 3, EPA) and docosahexaenoic acid (22:6 n = 3, DHA). Eicosanoids are synthesized rapidly in response to multiple factors (e.g. allergy, infection, and injury) and act putatively through their cognate receptors in local cells. Although some eicosanoids exhibit immediate and short-lasting activity, they play an important role in many chronic diseases, including asthma, allergy, autoimmune diseases, and malignancies since they have pleiotropic functions, such as pro-inflammation, anti-inflammation, vasodilation, analgesia, and hyperalgesia (Dennis and Norris, 2015; Sokolowska et al., 2020). Interestingly, some eicosanoids were found to have dual actions, for example, lipoxins, resolvins, and protectins, which have been extensively reported to be anti-inflammatory and pro-resolving (Serhan et al., 2008). Emerging evidence showed the dominant roles of metabolites of PUFAs involved in the regulation of inflammation, pain, angiogenesis, and cancer (Zhang et al., 2014a). The COX- and LOX-mediated PUFAs metabolism have been well documented in tumorigenesis and cancer progression (Wang and Dubois, 2006; Cathcart et al., 2012; Knab et al., 2014; Tuncer and Banerjee, 2015). However, the roles of the CYP pathway-mediated metabolites of PUFAs in the pathogenesis of cancer has not been fully studied.

CYP enzymes catalyze a variety of oxidative and some reductive reactions involving thousands of substrates (Guengerich, 2007). The substrates of CYPs encompass xenobiotics, including substances that occur biologically but are exogenous to humans, such as antibiotics and synthetic organic chemicals (Porter and Coon, 1991), and endogenous compounds, such as cholesterol, testosterone, progesterone, prostaglandin H_2_, corticosterone, retinoic acid, vitamin D_3_, and some PUFAs, like AA and LA (Guengerich, 2017). CYP enzymes can mediate the metabolisms of the lipophilic endogenous and xenobiotic compounds into hydrophilic or polar compounds, which could be excreted from the body easily (Chang and Kam, 1999). Firstly, the substrate binds to the active sites of CYP enzymes. Then the reductive reaction of the heme iron from a ferric to a ferrous is occurred by an electron transferred from a reduced NADPH (Chang and Kam, 1999). After that, the oxygen molecule temporarily binds at the heme-containing active site (Zanger and Schwab, 2013). At last, the substrate molecule is inserted by an oxygen atom, and water is formed by other relevant atoms simultaneously. Therefore, CYP monooxygenases incorporate one atom of oxygen into their substrates (Ortiz de Montellano, 2010). Here we focus on the CYP-derived metabolites of PUFAs and their multiple functions in cancer.

### The Cytochrome P450-Derived Eicosanoids of n-6 Polyunsaturated Fatty Acids

**FIGURE 1:**
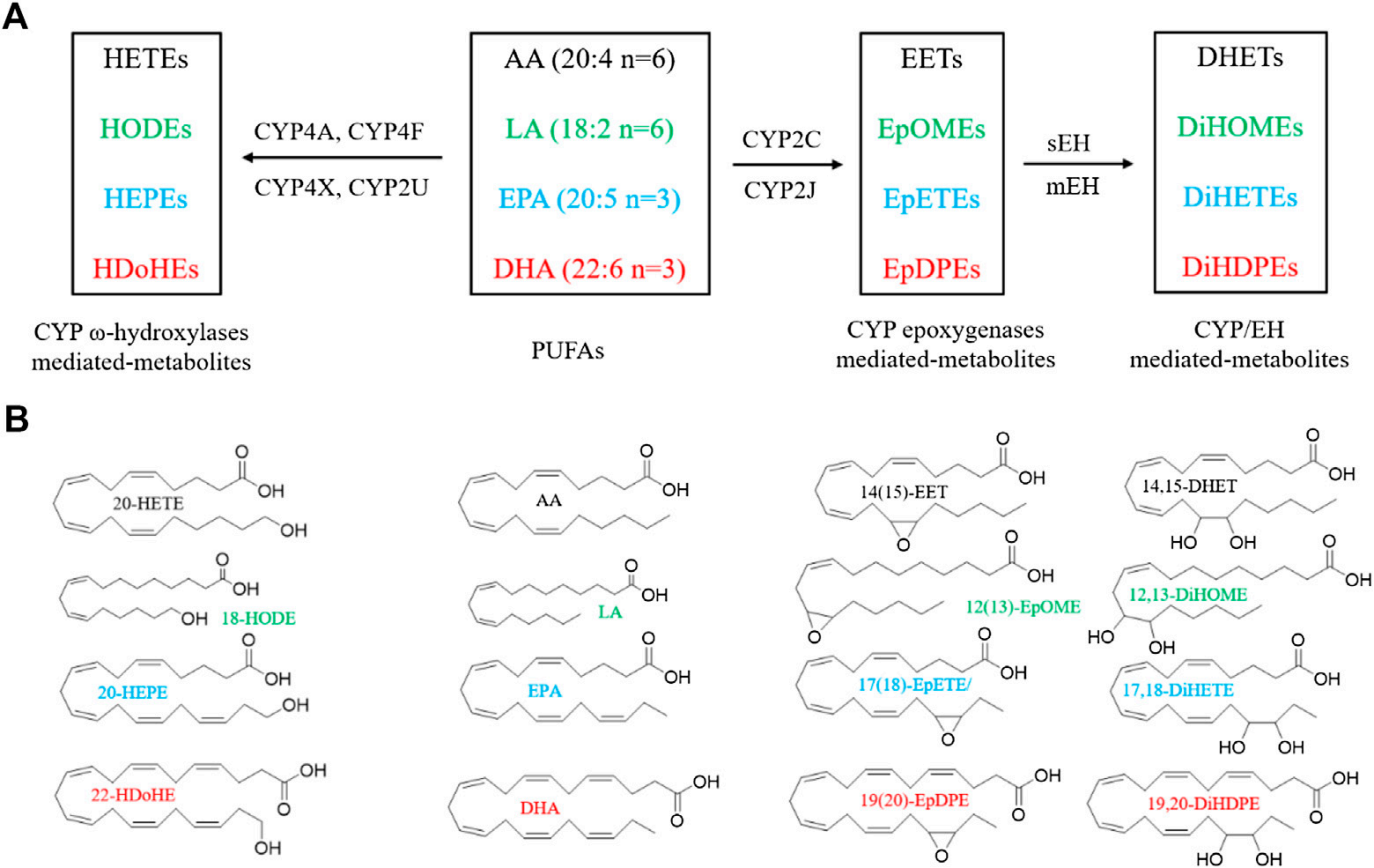
Metabolism of PUFAs by CYP enzymes. **(A)** A simplified cascade of PUFAs discussed in this paper. AA, arachidonic acid; LA, linoleic acid; EPA, eicosapentaenoic acid; DHA, docosahexaenoic acid; CYP, cytochrome P450; sEH, soluble epoxide hydrolase; DHET, dihydroxyeicosatrienoic acid; DiHOME, dihydroxy octadecamonoeneoic acid; DiHETE, dihydroxyeicosatetraenoic acid; DiHDTE, dihydroxydocosatetraenoic acid; EET, epoxyeicosatrienoic acid; EpOME, epoxyoctadecamonoeneoic acid; EEQ or EpETE, epoxyeicosatetreaenoic acid; EDP or EpDPE, epoxydocosapentaenoic acid; HDoHE, hydroxydocosahexaenoic acid; HEPE: hydroxyeicosapentaenoic acid; HETE, hydroxyeicosatetraenoic acid; HODE, hydroxyoctadecadienoic acid; mEH, microsomal epoxide hydrolase; sEH, soluble epoxide hydrolase. The metabolites are from the fatty acids in the same color. **(B)** The chemical structures of PUFAs and the representative metabolites.

CYPs consisting of 57 functional genes in human are a superfamily of enzymes which mediate the metabolism of exogenous and endogenous compounds (Jamieson et al., 2017; Nelson et al., 2004). AA and LA are the most common substrates of the CYP enzyme system. CYP enzymes relevant to AA metabolism include two main branches: the ω-hydroxylase and epoxygenase pathways (Panigrahy et al., 2010). Epoxygenases (mainly CYP2C and CYP2J isoforms) convert AA to epoxyeicosatrienoic acids (EETs), including 5(6)-, 8(9)-, 11(12)-, and 14(15)-EET (Wu et al., 1997). ω-hydroxylases (mainly CYP4A and CYP4F isoforms) convert AA to 19-, and 20-hydroxyeicosatetraenoic acids (HETEs) (Figure 1) (Panigrahy et al., 2010). In addition, CYP4X1 and CYP2U1 can metabolize AA to 19- and 20-HETE, as well as 8(9)-, and 14(15)-EET (Chuang et al., 2004; Stark et al., 2008). LA is the primary exogenous precursor of essential fatty acids, obtained from many diets. Within the body, LA can be catalyzed to the formation of 9(10)- and 12(13)-epoxyoctadecenoic acids (EpOMEs) in the presence of CYP epoxygenases. Both EETs and EpOMEs are metabolically unstable and can be rapidly metabolized to corresponding fatty acid diols, dihydroxyeicosatrienoic acids (DHETs), and dihydroxyoctadecenoic acid (DiHOMEs), respectively, by soluble epoxide hydrolase (sEH) and microsomal EH (mEH) (Figure 1) (Zhang et al., 2014a). Recently, the CYP/sEH eicosanoid pathway related to inflammation and cancer gains many interests in academic researches.

### The Cytochrome P450-Derived Eicosanoids of n-3 Polyunsaturated Fatty Acids

The n-3 PUFAs, mainly EPA and DHA, can be catalyzed by CYP isozymes into functional eicosanoids. EPA is metalized into ω/(ω-1)-hydroxyeicosapentaenoic acids (19- and 20-HEPE) by CYP ω-hydroxylases, and five regioisomeric epoxyeicosatetraenoic acids [5(6)-, 8(9)-, 11(12)-, 14(15)-, 17(18)-EEQ, or EpETE] by CYP epoxygenases (Figure 1) (Van Rollins et al., 1988). DHA can also be metalized into ω/(ω-1)-hydroxydocosahexaenoic acids (21- and 22-HDoHE) by CYP ω-hydroxylases, and six regioisomeric epoxydocosapentaenoic acids [4(5)-, 7(8)-, 10(11)-, 13(14)-, 16(17)-, 19(20)-EDP, or EpDPE), respectively, by CYP epoxygenases (Figure 1) (VanRollins et al., 1984). The epoxy metabolites EEQ and EDP can be further metabolized by sEH and mEH enzyme to the corresponding diols.

### The Presence and Location of Cytochrome P450s in Organs

The CYP superfamily comprises 57 functional CYP genes and 58 pseudogenes in humans (Nelson et al., 2004). The CYPs have been reported to express in all human tissues investigated (Porter and Coon, 1991). They are expressed predominately in the endoplasmic reticulum membrane, cell surface, and mitochondria (Neve and Ingelman-Sundberg, 2010), with the greatest abundance in the liver (Porter and Coon, 1991), small intestine (Thelen and Dressman, 2009), and kidney (Renaud et al., 2011).

In humans, CYP2C and CYP2J are the predominant epoxygenases that metabolize PUFAs. CYP2C and CYP2J are widely distributed in the human body, including but not limited to the cardiovascular system, kidney, lung, brain, gastrointestinal tract, cerebral cortex, hippocampus, fetal nasal mucosa, and many other tissues (Nelson et al., 2004). The CYP2C family is located on chromosome 10 and consists of at least seven genes and/or pseudogenes. The CYP2C8, CYP2C9, CYP2C18, and CYP2C19 are most involved in the metabolism of PUFAs. They are also convinced to be involved in the progression of a malignant tumor (Xu et al., 2011). Besides the above-mentioned four CYP2C enzymes, CYP2J2 is another epoxygenase acting as a regulator that catalyzes the metabolism of PUFAs. In the human body, CYP2J2 is mainly distributed in cardiovascular tissues, such as cardiomyocytes, coronary endothelial cells, and aorta and vein of coronary smooth muscle cells. Liver enzymes account for only 1–2% of total CYP in the liver, jejunum, ileum, and colon. Limited by its content, CYP2J2 generally does not play a decisive role in exogenous metabolism in theory. However, CYP2J2 has been found to play a dominant role in the intestinal metabolism of certain drugs, such as antihistamines, terfenadine, and ebastine. In the kidney, CYP2J2 is expressed in proximal convoluted tubule and collecting tubule. In addition to the expression in tissues such as the heart, kidney, and liver, CYP2J2 is also highly expressed in the cerebral cortex, frontal lobe, and hippocampus. Dutheil et al. showed that a variety of CYPs are distributed in the human brain, and the expression level of CYP2J2 is about 20% of the total content of CYPs. Moreover, Ahmed E. et al. reported that CYP2J2 was expressed in the pancreatic islets, consistent with the finding that EETs, the metabolic products of AA, regulate the levels of insulin and glucagon (Zeldin et al., 1997). CYP2J2 and CYP2C9 enzymes are co-expressed in the pituitary gland, suggesting they can regulate the release of pituitary hormones including prolactin and growth hormone most likely via EETs (Snyder et al., 1989; Irusta et al., 2007).

In mammals, the CYP4 family primarily mediates the ω-hydroxylation of PUFAs, which includes 12 genes and 13 enzymes, such as, CYP4A, CYP4B, CYP4F, CYP4V, CYP4X, and CYP4Z. CYP4A, CYP4B, CYP4X, and CYP4Z are located on chromosome 1, while CYP4F and CYP4V on chromosome 19 and 4, respectively (Nelson et al., 2004). The CYP4 is the largest one in the human CYP family, only a few of which mediate the ω-hydroxylation of PUFAs (Simpson, 1997). In the CYP4 family, CYP4A11, CYP4F2, CYP4F3A, and CYP4F3B are the most studied. CYP4A11 has been reported to express primarily in the liver and kidney, which can be regulated by peroxisome proliferator-activated receptor-α (PPARα) and catalyze the metabolism of AA and lauric acid. CYP4F2 is also mainly expressed in the liver and kidney, and it is regulated by the sterol regulatory element-binding protein (SREBP). The P450 gene CYP4F3 is unusual, and CYP4F3A and CYP4F3B are two different spliceosomes. CYP4F3A is expressed in neutrophils and CYP4F3B is primarily expressed in the human liver and kidney. They are both the main ω-hydroxylases of long-chain PUFAs. In addition, CYP4F8, CYP4F22, and CYP4V2 have been found to express predominantly in extrahepatic tissues. Among these enzymes, only CYP4V2 exhibits fatty acid ω-hydroxylase activity. CYP4X1 and CYP4Z1 are both extrahepatic CYPs, the former is highly expressed in the brain, skin, and airways, and is inducible by glucocorticoids and progesterone.

Other CYPs, such as CYP1A1, CYP 1A2, CYP1B1, CYP2D6, and CYP3A4, can detoxificate carcinogens regardless of whether they are expressed in the liver or kidney.

In addition to many studies investigating the expression of CYP epoxygenases in multiple organ tissues, a few studies summarized the expression of CYP epoxygenases in some specific cell types. Almost all the CYP epoxygenases were found in peripheral blood cells, vascular endothelial cells, and vascular smooth muscle cells (Xu et al., 2013; Sausville et al., 2019). In addition, CYP2J was reported to express in many cells, including but not limited to LS-174, ScaBER, SiHa, U251, A549, Tca-8113, Ncl-H446, HepG2, K562, HL-60, MOLT-4, Jurkat, Raji, autonomic ganglion nerves, and smooth muscle cells, pancreatic islet cells, Purkinje cells (DeLozier et al., 2007; Xu et al., 2013; Sausville et al., 2019).

### The Association of Polymorphisms of Cytochrome P450s With Cancers

Recently, the expression of various CYP genes has been proved to be closely related to malignant tumors. Polymorphisms of CYPs have been suggested to influence susceptibility to cancers for many years. Genetic polymorphisms in CYPs have been reported to be associated with individuals variations in drug metabolism and disease susceptibility (Pikuleva and Waterman, 2013; Mittal et al., 2015). Here, we discuss some important polymorphisms of CYPs in cancers **(** Table 1 **)**.

### CYP1A1

CYP1A1 is a hepatic and extrahepatic enzyme that is regulated by the aryl hydrocarbon receptor signaling pathway. It has been always associated with the metabolism of pro-carcinogenic compounds to highly carcinogenic metabolites. For CYP1A1, four common variants (T3801C, A2455G, T3205C, and C2453A) were widely studied for the susceptibility to various cancers (Bozina et al., 2009). T3801C (rs4646903) and T3205C are situated in the 3′ noncoding region while A2455G (rs1048943) and C2453A are ascertained in exon 7, which results in the transition of isoleucine to valine on codon 462 and threonine to asparagine on codon 461, respectively (Li et al., 2004). The monomorphism of T3205C locus was reported in Indians (Singh et al., 2007), Americans (San Francisco) (Hirata et al., 2008), and Northeast Thai women (Wongpratate et al., 2020). Among several populations, the polymorphisms of T3801C and/or A2455G were reported to associate significantly with the increased risk in cervical cancer (Juarez-Cedillo et al., 2007; Li et al., 2016; Jain et al., 2017; Wang et al., 2017; Ding et al., 2018). An association of T3801C (CC) genotype with increased cervical cancer risk was reported among the Asians population by a meta-analysis study (Wu et al., 2013). However, there is a lack of significant association between T3801C and A2455G polymorphisms and cervical cancer risk in Chinese, Japanese, Israeli Jewish, Polish, Indian, and Thai populations (Sugawara et al., 2003; Gutman et al., 2009; Roszak et al., 2014; Tan et al., 2016; Wongpratate et al., 2020). The A allele of C2453A is associated with the risk of lung cancer (Gallegos-Arreola et al., 2008; Ezzeldin et al., 2017), laryngeal squamous cell carcinoma (Gajecka et al., 2005), thyroid cancer (Siraj et al., 2008), and cervical cancer (Wongpratate et al., 2020), but not associated with breast (Li et al., 2004; Singh et al., 2007; Amrani et al., 2016), colorectal (Little et al., 2006), or gastric cancer (Agudo et al., 2006).

### CYP1A2

CYP1A2, which is similar to CYP1A1, can metabolize a broad range of foreign compounds and drugs. An SNP C>A (rs762551) was found in intron one of CYP1A2 (Womack et al., 2012), which influences the inducibility of CYP1A2 (Koonrungsesomboon et al., 2018). The highest CYP1A2 induction rate was reported in AA genotype (Sachse et al., 1999), and the high enzyme activity carriers were at high risk of lung cancer (Seow et al., 2001; Bu et al., 2014). On the other hand, the low activity or downregulation of CYP1A2 influenced by the SNP would result in the progression of hepatocellular carcinoma (HCC). As the substrate of CYP1A2, 17 β-estradiol can be metabolized to 2-hydroxyestradiol which is then converted to 2-methoxyestradiol that inhibits HCC cells proliferation by inducing apoptosis (Ren et al., 2016). More recently, the CYP1A2 SNP rs762551 was found to be significantly associated with the high risk in breast cancer in the Jordanian population (Al-Eitan et al., 2019).

### CYP1B1

CYP1B1 not only mediates the metabolisms of xenobiotics, e.g. theophylline, ethoxyresorufin, and caffeine (Rochat et al., 2001; Zanger and Schwab, 2013) but also activates some procarcinogens, such as aromatic amines, heterocyclic amines, nitropolycyclic and polycyclic hydrocarbons (Chun and Kim, 2016). CYP1B1 mutations were a causative factor of diseases. For example, L432V and A119S (rs1056827) polymorphisms of the CYP1B1 gene were reported to increase the risk of developing endometrial cancer (Zhu et al., 2011) and laryngeal cancers (Yu et al., 2015).

### CYP2A6

CYP2A6 is an essential hepatic enzyme involved in the metabolism of drugs, is responsible for a major metabolic pathway of nicotine. The first polymorphism identified of CYP2A6 was a nonsynonymous polymorphism (L160H) (rs1801272) which leads to no enzyme activity (Fernandez-Salguero et al., 1995). There are more than 30 non-synonymous polymorphisms in nine exons (Di et al., 2009). Some polymorphisms were found to be associated with smoking behavior, drug metabolism, and lung cancer risk (Di et al., 2009). The wild type CYP2A6*1A is with normal enzyme activity, and the CYP2A6*4, including CYP2A6*4A, CYP2A6*4B, and CYP2A6*4D, have no enzyme activity (Hukkanen et al., 2005). The CYP2A6*5 encoded an unstable enzyme activity since the substitution of Glycin-479 by valine occurred (Oscarson et al., 1999). CYP2A6 can activate procarcinogens, for instance, nitrosamines and aflatoxins. The absence of the CYP2A6 enzyme could reduce the risk of lung cancer because the activation of procarcinogens would be decreased. Therefore, the phenotypes of CYP2A6*4 and CYP2A6*5 protect the carriers against lung cancer or other cancers (Raunio et al., 2001).

### CYP2C9

CYP2C9 metabolizes about 15% of clinically administrated drugs. Two common non-synonymous polymorphisms of CYP2C9, R144C, rs1799853 (CYP2C9*2), and I359L, rs1057910 (CYP2C9*3), have been reported to be highly frequent in Caucasian populations (Sistonen et al., 2009; Van Booven et al., 2010). These two polymorphisms result in poor metabolic activity of CYP2C9 (Van Booven et al., 2010), and are positively associated with the risk of cancer. Individuals with CYP2C9*2 (R144C, rs1799853) polymorphism have a several-fold increased risk of head and neck squamous cell carcinoma (HNSSC) (Yadav et al., 2014). On the contrary, as the CYP2C9 (R144C, rs1799853, and I359L, rs1057910) variants metabolize AA less efficiently than CYP2C9 wild type, they were proved to retard the development of non-small cell lung cancer (NSCLC) due to the reduced ability to generate EETs (Sausville et al., 2018). Recently, the relationship of CYP2C9 polymorphism with colorectal cancer (CRC) susceptibility was investigated by a number of case-control studies. But the results were controversary. A meta-analysis of 13 studies involving a total of 20,879 subjects for CYP2C9 (R144C, rs1799853 and I359L, rs1057910) polymorphisms to evaluate the effect of CYP2C9 on genetic susceptibility for CRC suggest that the CYP2C9 (R144C, rs1799853 and I359L, rs1057910) polymorphisms are not associated with CRC susceptibility (Zhao et al., 2013). Also, the CYP2C9 (R144C, rs1799853, and I359L, rs1057910) polymorphisms are not associated with lung cancer risk among African-Americans and Caucasians in Los Angeles (Zhao et al., 2013) or in white Spanish (Garcia-Martin et al., 2002).

### CYP2C19

Among the CYP2C subfamily, CYP2C19 is the most polymorphic (Lee, 2012). CYP2C19 polymorphism leads to differences in enzyme expression and metabolic activity between individuals. CYP2C19 polymorphisms classified the population to poor, extensive, and ultra-rapid metabolic activity (Reynald et al., 2012). The two primary point mutation sites of CYP2C19 are CYP2C19*2 and CYP2C19*3, which cause poor metabolizer phenotype of CYP2C19. CYP2C19 polymorphisms have been analyzed about the prostate, bladder, lung, liver, colorectal cancer, and other cancers. No association of the CYP2C19*2 allele and prostate cancer was identified in the Swedish and Danish population (Wadelius et al., 1999). But there is a weak association between the CYP2C19*2 allele and bladder cancer (Brockmoller et al., 1996). Yan et al. found that there was a significant interaction between CYP2C19*3 and smoking in increasing the risk of lung cancer in a Chinese population (Yan et al., 2014). Similar results were reported in the Japanese population (Tsuneoka et al., 1996). CYP2C19*3 was identified to be associated with breast cancer risk in women (Gan et al., 2011). In contrast, a decreased breast cancer risk for carriers of the CYP2C19*17 allele was observed in German women (Justenhoven et al., 2009), and a meta-analysis has found that CYP2C19*2 and CYP2C19*17 genotypes are associated with increased survival of breast cancer patients treated with tamoxifen (Bai et al., 2014). Zhou et al. found that CYP2C19*2 causes a poor metabolizer phenotype, while CYP2C19*3 is associated with the increased risk of digestive system cancer, especially in East Asians (Zhou et al., 2013). Moreover, poor metabolizer genotypes were found to be associated with the increased risks in many cancers, such as esophagus cancer, gastric cancer, lung cancer, head neck cancer, and hepatocellular carcinoma, suggesting the CYP2C19*2 and CYP2C19*3 most likely contributes to cancer susceptibility, particularly in the Asian populations (Wang et al., 2013).

### CYP2D6

CYP2D6 is one of the most studied enzymes in the field of pharmacogenetics. It exhibits large interindividual variability on drug metabolism. The polymorphism of CYP2D6 causes different metabolizer genotypes, including poor, intermediate, efficient, or ultra-rapid ones (Ingelman-Sundberg, 2005). The number of CYP2D6 polymorphisms is over seventy-five, and the association between CYP2D6 polymorphisms and cancer risk has been studied for many years (Agundez, 2004). Over twenty years ago, London et al. reported that the presence of inactivating CYP2D6 alleles (CYP2D6*4, CYP2D6*3, CYP2D6*5, and CYP2D6*16) may decrease the risk of lung cancer among the African-Americans, suggesting the CYP2D6 genetic polymorphism is not the strong risk factor for lung cancer but may play a minor role (London et al., 1997). By a meta-analysis, a minor but statistically significant association of CYP2D6 polymorphism with lung cancer susceptibility was established (Rostami-Hodjegan et al., 1998). Recently, associations between childhood acute leukemia (CAL) and genetic polymorphism of CYP2D6*4 for homozygous alleles were reported, suggesting CYP2D6*4 polymorphism could play a vital role in the etiology of CAL (Ferri et al., 2018). The association of Liver cancer with CYP2D6 genotype was also established by Agundez et al. (Agundez et al., 1995) In the HCC patients, the CYP2D6*10 allelic frequency was significantly different from those of control subjects. CYP2D6*10 is also suggested to be a potential biomarker for hepatocarcinogenesis risk (Zhou et al., 2016). The role of CYP2D6 polymorphism in melanoma has been investigated by different research groups with consistent results, indicating individuals with defect genes are at increased risk (Wolf et al., 1992; Dolzan et al., 1995; Strange et al., 1999). In other cancers, CYP2D6 polymorphisms were demonstrated to be associated with prostate (Wadelius et al., 1999; Sobti et al., 2006), bladder (Abdel-Rahman et al., 1997; Ouerhani et al., 2008) and renal cancers (Ahmad et al., 2013).

### CYP3A4

CYP3A4 is the best-studied gene polymorphism in the CYP3A family. A meta-analysis comprising 55 separate studies including 22,072 cancer cases and 25,433 controls found a significant association between CYP3A4*1B and cancer risk especially leukemia in the overall population (Zheng et al., 2018). In the Chinese Han population, a relationship between the TT genotype of CYP3A4*1G (rs2242480) polymorphism and the risk of breast cancer was established (Liu et al., 2019). He et al. found that CYP3A4 A392G polymorphism, but not CYP3A5 Met235Thr, is associated with the increased risk in prostate cancer among Caucasians (He et al., 2014). Although there are many studies on CYP3A4 gene polymorphism and cancer susceptibility, the underlying mechanism is still unclear and needs to be further investigated.

## The Function of Cytochrome P450-Mediated Eicosanoids in Cancers

### The Roles of Epoxyeicosatrienoic Acids in Cancer

EETs were discovered in the early 1980s (Capdevila et al., 1981a; Capdevila et al., 1981b; Chacos et al., 1982). EETs have been found to function as the regulators of cardiac, vascular (Fleming, 2007; Fleming, 2008; Fleming, 2011; Fleming, 2016), and renal physiology (Imig, 2005; Capdevila and Wang, 2013; Capdevila et al., 2015), indicating many important roles on the homeostasis of healthy tissues. Nakagawa et al. reported that the loss of EETs is associated with hypertension (Nakagawa et al., 2006). More recently, EETs have also been found to be associated with tumorigenesis, cancer metastasis, and angiogenesis (Jiang et al., 2005; Jiang et al., 2007; Pozzi et al., 2007; Pozzi and Capdevila, 2008; Yang et al., 2009; Mitra et al., 2011; Panigrahy et al., 2012), recovery of cardiac tissue from ischemic insult (Seubert et al., 2007) and other pathophysiologic processes.

CYP-mediated biosynthesis of four EETs [5(6)-, 8(9)-, 11(12)-, and 14(15)-EET] has been implicated in tumor growth and angiogenesis, as well as suppression of inflammation in murine models of cancers (Panigrahy et al., 2011). Compared to adjacent normal tissue, human breast cancer tissue has a higher level of 14 (15)-EET, which was due to increased CYP2C8, CYP2C9, and CYP2J2 and decreased sEH (Wei et al., 2014). Overexpression of CYP2J2 was found to be overexpressed in human neoplastic tissue and human cell lines when compared with the adjacent normal tissue and normal cell lines, respectively (Jiang et al., 2005; Jiang et al., 2007). CYP2C9 was found to express in the vasculature of several human tumor samples and be the regulatory target of human peroxisomal proliferator-activated receptor-alpha (PPARα), which have anti-angiogenic and anti-tumorigenic properties (Pozzi et al., 2010). The elevated EET levels by CYP overexpression or directly provided by the pump showed the ability to promote cancer metastasis in a murine model of cancer (Panigrahy et al., 2012). 14 (15)-EET has been exhibited to promote the proliferation of vascular endothelial cells (Cheranov et al., 2008) and estrogen receptor-positive breast cancer epithelial cells (Mitra et al., 2011). The molecular mechanism underlying the function of EETs on cancer cell proliferation is partly through activation of the PI_3_-kinase/AKT pathway and the STAT_3_ pathway (Jiang et al., 2005). More recently, Guo et al. discovered the mechanisms by which cancer cell-intrinsic CYP monooxygenases promote tumor progression are associated with breast cancer mitochondria and EETs promoted the electron transport chain/respiration and inhibited AMPKα (Guo et al., 2017). In triple-negative breast cancer (TNBC), EETs are important metastasis drivers. EET concentrations are associated with the upregulation of CYP2C19 and CYP2J2 (Apaya et al., 2019). In addition, EETs promote epithelial-mesenchymal transition (EMT) and resistance via the STAT and AKT signaling pathways (Zhang et al., 2006; Jiang et al., 2007; Mitra et al., 2011; Wei et al., 2014; Luo et al., 2018).

**FIGURE 2:**
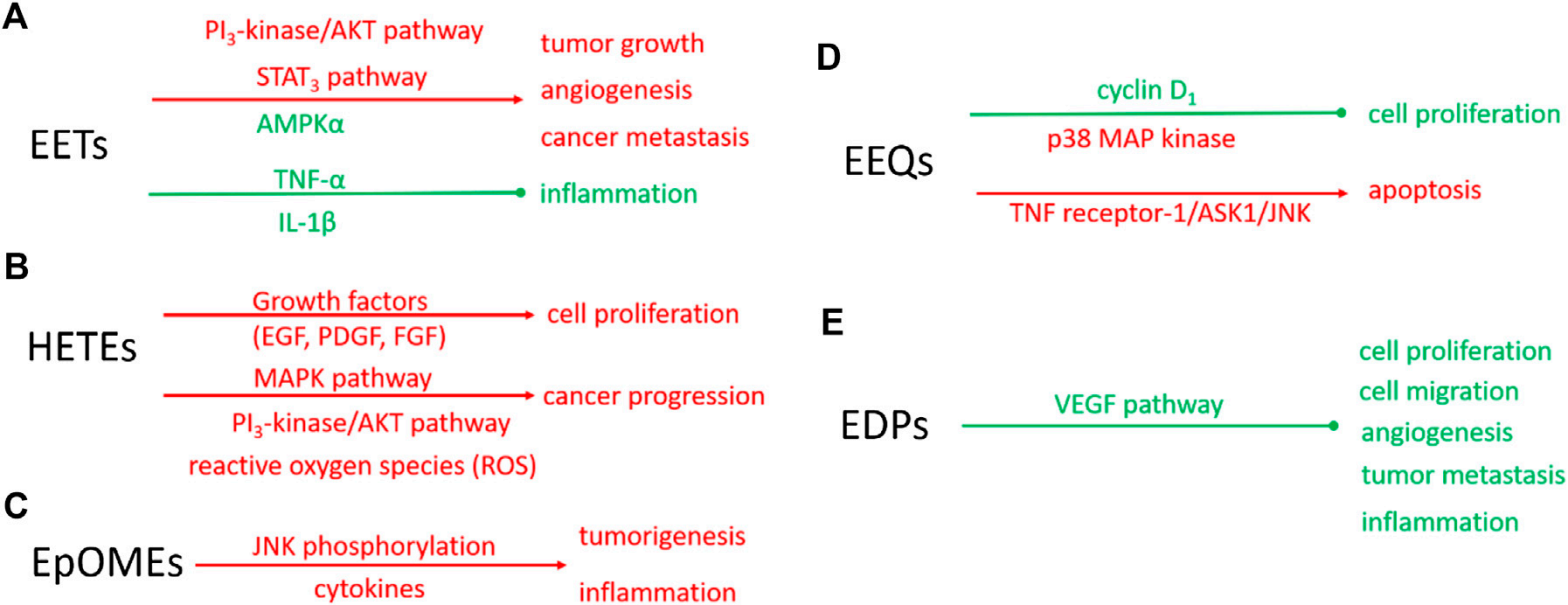
Schematic diagram of molecular mechanisms of eicosanoids on the biological function of tumors. Red: indicating the enhanced signaling pathway or biological function; Green: indicating the suppressed signaling pathway or biological function solid line: proven pathways. The molecular mechanisms of EETs **(A)**, HETEs **(B)**, EpOMEs **(C)**, EEQs **(D)**, and EDPs **(E)**.

EETs are also anti-inflammatory (Node et al., 1999), which makes the biological action of EETs on cancer more complicated. In the murine model, 11(12)-EET can decrease the adhesion of mononuclear to vascular endothelium induced by tumor necrosis factor-α (TNF-α) (Node et al., 1999). 14 (15)-EET can also inhibit the expression of TNF-α and IL-1β induced by LPS in mouse macrophages (Zhang et al., 2013b). These results supported that EETs are anti-inflammatory in human and mouse tissues. However, whether and how EETs play an anti-inflammatory role in cancer cells needs to be further investigated. The possible mechanisms underlying EETs regulate cancers are summarized in Figure 2A.

Recently, EETs were discovered to act the biological function in a receptor-dependent manner. However, the receptors of EETs have not been identified. Some research suggested that the actions of EETs are in part mediated via G protein-coupled receptor (GPCR) signaling. Five G protein-coupled receptors of the prostaglandin receptor family (PTGER2, PTGER4, PTGFR, PTGDR, and PTGER3IV) may be the EET receptors (Liu et al., 2017).

### The Roles of Hydroxyeicosatetraenoic Acids in Cancer

The studies of the role of HETEs in cancer focus on 20-HETE. 20-HETE formed by CYP4 enzymes exhibits the proinflammatory function (Khanapure et al., 2007). In recent years, more attention has been paid to the promoting role of 20-HETE in cancer progression (Panigrahy et al., 2010; Alexanian and Sorokin, 2013). The endogenous 20-HETE formation has been implicated in cell proliferation by growth factors, including epidermal growth factor (EGF), platelet-derived growth factor (PDGF), and fibroblast growth factor (FGF). Guo et al. found CYP4/20-HETE pathway could influence the tumor volume. When implantation of glioma cells transfected with CYP4A1 into the rat, the tumor volume is a 10-fold increase compared with normal cells (Guo et al., 2008). In NSCLC cells, CYP4A/20-HETE increased the tumor growth rate and metastasis (Yu et al., 2011). The signaling mechanisms in CYP4/20-HETE induced cancer progression are mainly about the activation of MAPK pathway (Muthalif et al., 1998), PI_3_K/Akt (Yu et al., 2011), and reactive oxygen species (ROS) (Guo et al., 2007), which was summarized as Figure 2B.

### The Roles of Epoxyoctadecenoic Acids in Cancer

EpOMEs are the major epoxygenated fatty acids in human plasma produced from LA. EpOMEs ， also called leukotoxins, have been shown to act as a responsible factor in circulatory shock, burn, pulmonary edema, and inflammation (Hanaki et al., 1991; Hayakawa et al., 1990; Kosaka et al., 1994; Ozawa et al., 1988; Totani et al., 2000). EpOMEs are pro-inflammatory in severe burn patients (Totani et al., 2000; Zheng et al., 2001). Recently, EpOMEs are found to have pro-cancer activity in a murine model of colorectal cancer (Wang et al., 2019). Evidence indicated that high LA diets increased the azoxymethane-induced colon tumorigenesis in rat models (Zheng et al., 2001; Wu et al., 2004; Fujise et al., 2007; Enos et al., 2016). Case-control studies showed opposing associations of serum n-3 and n-6 PUFAs with the risk of colorectal adenoma (Pot et al., 2008). Researchers found treatment with 12(13)-EpOME increased cytokine production and JNK phosphorylation *in vitro* and exacerbated azoxymethane (AOM)/dextran sodium sulfate (DSS)-induced colon tumorigenesis *in vivo* (Wang et al., 2019). The underlying molecular mechanisms involved in the regulatory role of EpOMEs on cancer progression are not fully understood, partly because the specific receptors or direct cellular targets of EpOMEs are unknown. The effects of EpOMEs on cancers were summarized as Figure 2C. EpOMEs could be further metabolized to form DiHOMEs in the presence of sEH. Studies also showed DiHOMEs could induce chemotaxis, tissue injury, and cause mortality like EpOMEs in animal models (Moghaddam et al., 1997; Zheng et al., 2001).

### The Roles of Epoxyeicosatetraenoic Acid in Cancer

Epidemiological studies revealed that dietary intake of EPA, the precursor of EEQs, decreases cancer risk (Bougnoux, 1999). Cui et al. have found that 17(18)-EEQ, but not 14(15)-, 11(12)-, 8(9)-, 5(6)-EEQ suppressed cell proliferation by down-regulating cyclin D_1_ and activation of growth-suppressing p38 MAP kinase (Cui et al., 2011). Another research also revealed that a novel synthetic analog of 17(18)-EEQ activated TNF receptor-1/ASK1/JNK signaling to promote apoptosis in human breast cancer cells, manifesting the anticancer action of 17(18)-EEQ (Dyari et al., 2017). Up to now, the role of EEQ in cancer and its molecular mechanism has not been fully studied, which needs to be further studied in the future. The putative mechanism underlying EEQ in cancer was summarized as Figure 2D.

### The Roles of Epoxydocosapentaenoic Acids in Cancer

EDPs are the metabolites of DHA mediated by CYP enzymes. They are suggested to be responsible for some of the beneficial effects of n-3 PUFAs and n-3 PUFA-rich diet (Arnold et al., 2010). Evidence has shown that the metabolites of n-3 PUFAs, mainly EDPs, mediate some effects in chronic disease conditions, such as hypertension, pain, and kidney diseases (Ulu et al., 2014; Deng et al., 2017; Hasegawa et al., 2017). More recently, some experimental studies showed that 16(17)- and 19(20)-EDPs are important mediators in suppressing inflammation and inhibiting angiogenesis, endothelial cell migration, endothelial cell proliferation, and tumor metastasis (Zhang et al., 2013a; Yanai et al., 2014; Hasegawa et al., 2017). EDPs are super unstable *in vivo* since they could be rapidly metabolized by sEH to form corresponding diols. Zhang et al. found 19,20-dihydroxydocosapentaenoic acid (19,20-DiHDPA), the metabolite of 19 (20)-EDP mediated by sEH, did not have any effect on tumor growth, indicating that the anticancer effect was from 19 (20)-EDP but not its diol metabolite (Zhang et al., 2013a). The putative mechanism of EDPs in cancer was summarized as Figure 2E.

## Conclusion

Increasing studies supported the important role of CYP-derived eicosanoids in the progression of cancer and the resolution of inflammation. The action of EETs and EpOMEs has been investigated for decades while EEQs and EDPs are less-studied. Further studies are suggested to pay more attention to EEQs and EDPs since increasing the supplement of EPA and DHA with a focus on the biological activities of these eicosanoids and underlying mechanisms.

The additional complexity of the regulation of eicosanoids on inflammation, pain, angiogenesis, and cancer is the sEH enzyme, which can rapidly metabolize many CYPs-derived eicosanoids to corresponding fatty acid diols (Chacos et al., 1983; Zeldin et al., 1995; Zhang et al., 2014a). sEHs may modulate tumor angiogenesis by hydrolyzing pro-angiogenic EETs (Panigrahy et al., 2012). sEH also can hydrolyze anti-angiogenic epoxides of DHA (Zhang et al., 2013a). Co-inhibition of sEH resulted in a synergistic anti-inflammatory effect of the inhibitors of COXs and LOXs (Schmelzer et al., 2006; Liu et al., 2010; Hwang et al., 2011). Zhang et al. reported the anti-cancer effects of a dual inhibitor of sEH and COX (Zhang et al., 2014b). In short, the roles of CYPs/epoxides/sEH axis in cancer progression is substrate-dependent. Generally speaking, the CYP-mediated eicosanoids derived from ω-6 fatty acids (e.g. LA and AA) exacerbate cancer while the ones from ω-3 fatty acids (e.g. EPA and DHA) are beneficial in the prevention and/or treatment of cancer. Manipulation of the CYPs/sEH pathway with or without supplement of ω-3 fatty acids to regulate target eicosanoids levels may be a promising strategy to prevent and/or treat cancers.

**TABLE 1:** Examples of some cancers associated with cytochrome P450 enzymes.

CYPs	Polymorphism	Cancers	population	References
CYP1A1	T3801C	Cervical cancer	Asians	Wu et al., 2013
	(rs4646903)		Caucasians and Asians	Ding et al., 2018
	and/or		Indians	Jain et al., 2017
	A2455G		Mexican	Juarez-Cedillo et al., 2007
	(rs1048943)		Chinese	Li et al., 2016
	—		Caucasians	Wang et al., 2017
	C2453A	Lung cancer	Egyptian	Ezzeldin et al., 2017; Gallegos-Arreola et al., 2008
			Mexican	
	—	Laryngeal squamous cell carcinoma	Caucasian	Gajecka et al., 2005
		Thyroid cancer	Middle eastern	Siraj et al., 2008
		Cervical cancer	Northeast Thai	Wongpratate et al., 2020
	T3205C	Breast cancer	Indians	Singh et al., 2007
	(rs1800031)	Endometrial cancer	Caucasian	Hirata et al., 2008
	—	Cervical cancer	Northeast Thai	Wongpratate et al., 2020
CYP1A2	rs762551	Lung cancer	Caucasians	Bu et al., 2014
		Breast cancer	Jordanian	Al-Eitan et al., 2019
CYP1B1	rs1056827	Endometrial cancer	Chinese	Zhu et al., 2011
		Laryngeal cancer	Chinese	Yu et al., 2015
CYP2A6	CYP2A6*4	Lung cancer	Japanese	Raunio et al., 2001
	and		Caucasian	
	CYP2A6*5		—	
CYP2C9	rs1799853	Head and neck squamous cell carcinoma	Indian	Yadav et al., 2014
	rs1799853	Non-small cell lung cancer	Caucasians	Sausville et al., 2018
	and			
	rs1057910			
CYP2C19	CYP2C19*2	Bladder cancer	German	Brockmoller et al., 1996
	(rs4244285)			
	CYP2C19*3	Lung cancer	Chinese	Yan et al., 2014
	—		Japanese	Tsuneoka et al., 1996
		Breast cancer	Chinese	Gan et al., 2011
		Digestive system cancer	East Asians	Zhou et al., 2013
	CYP2C19*17	Breast cancer	German	Justenhoven et al., 2009
	(rs12248560)			
CYP2D6	CYP2D6*1A	Bladder cancer	Egyptian	Abdel-Rahman et al., 1997
	CYP2D6*3	Lung cancer	African-Americans	London et al., 1997
	CYP2D6*4			
	CYP2D6*5			
	and CYP2D6*16			
	CYP2D6*4	Childhood acute leukemia	Italian	Ferri et al., 2018
		Malignant melanoma	British	Strange et al., 1999
	CYP2D6*3	Prostate cancer	Danish	Wadelius et al., 1999
	and			
	CYP2D6*4			
	CYP2D6*10	Hepatocellular carcinoma	Chinese	Zhou et al., 2016
	(rs10655852)			
CYP3A4	CYP3A4*1B	Leukemia	Chinese	Zheng et al., 2018
	CYP3A4*1G	Breast cancer	Chinese	Liu et al., 2019
	(rs2242480)			
	CYP3A4 A392G	Prostate cancer	Caucasians	He et al., 2014

## Author Contributions

YL and JYL designed the paper frame; YL wrote the draft; JYL critically revised and finalized the paper. YL and JYL approved the final version.

## Conflict of Interest

The authors declare that the research was conducted in the absence of any commercial or financial relationships that could be construed as a potential conflict of interest.

## Abbreviations

**Abbreviations:** AA, arachidonic acid; ALA, α-linolenic acid; ALL, acute lymphoblastic leukemia; AOM/DSS, azoxymethane/dextran sodium sulfate; CML, chronic myelogenous leukemia; COX, cyclooxygenase; CRC, colorectal cancer; CYP, cytochrome P450; DHA, docosahexaenoic acid; DHET, dihydroxyeicosatrienoic acid; DiHOME, dihydroxyoctadecenoic acid; EDP or EpDPE, epoxydocosapentaenoic acid; EEQ or EpETE, epoxyeicosatetraenoic acid; EET, epoxyeicosatrienoic acid; EGF, epidermal growth factor ； EMT, epithelial-mesenchymal transition; EPA, eicosatetraenoic acid; EpOME, epoxyoctadecenoic acid; FGF, fibroblast growth factor; GIST, gastrointestinal stromal tumors; GLA, γ-linolenic acid; HCC, hepatocellular carcinoma; HDoHE, hydroxydocosahexaenoic acid; HEPE, hydroxyeicosapentaenoic acid; HETE, hydroxyeicosatetraenoic acid; HNSCC, head and neck squamous cell carcinoma; LA, linoleic acid; LOX, lipoxygenase; mEH, microsomal epoxide hydrolase; NSCLC, non-small cell lung cancer; PDGF, platelet-derived growth factor; PLA_2_, phospholipase A_2_; PPAR, peroxisome proliferator-activated receptor; PUFA, polyunsaturated fatty acid; RA, retinoic acid; sEH, soluble epoxide hydrolase; SREBP, sterol regulatory element binding protein; TNBC, triple-negative breast cancer; TNFα, tumor necrosis factor-α.
